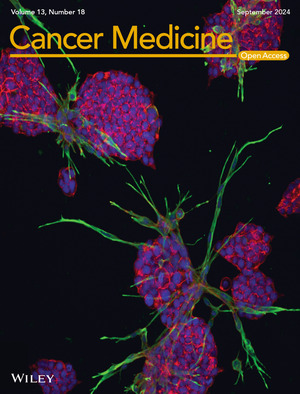# Cover Image

**DOI:** 10.1002/cam4.70306

**Published:** 2024-10-08

**Authors:** Syeda Afshan, Yu Gang Kim, Jesse Mattsson, Malin Åkerfelt, Pirkko Härkönen, Martin Baumgartner, Matthias Nees

## Abstract

The cover image is based on the article *Targeting the cancer cells and cancer‐associated fibroblasts with next‐generation FGFR inhibitors in prostate cancer co‐culture models* by Syeda Afshan et al., https://doi.org/10.1002/cam4.70240.